# Realization of a vertical topological p–n junction in epitaxial Sb_2_Te_3_/Bi_2_Te_3_ heterostructures

**DOI:** 10.1038/ncomms9816

**Published:** 2015-11-17

**Authors:** Markus Eschbach, Ewa Młyńczak, Jens Kellner, Jörn Kampmeier, Martin Lanius, Elmar Neumann, Christian Weyrich, Mathias Gehlmann, Pika Gospodarič, Sven Döring, Gregor Mussler, Nataliya Demarina, Martina Luysberg, Gustav Bihlmayer, Thomas Schäpers, Lukasz Plucinski, Stefan Blügel, Markus Morgenstern, Claus M. Schneider, Detlev Grützmacher

**Affiliations:** 1 Forschungszentrum Jülich GmbH, Peter Grünberg Institute (PGI-6) and JARA-FIT, 52425 Jülich, Germany; 2 Faculty of Physics and Applied Computer Science, AGH University of Science and Technology, al. Mickiewicza 30, 30-059 Kraków, Poland; 3 RWTH Aachen University, II Institute of Physics B and JARA-FIT, 52074 Aachen, Germany; 4 Forschungszentrum Jülich GmbH, Peter Grünberg Institute (PGI-9) and JARA-FIT, 52425 Jülich, Germany; 5 Forschungszentrum Jülich GmbH, Ernst Ruska-Centre for Microscopy and Spectroscopy with Electrons, 52425 Jülich, Germany; 6 Forschungszentrum Jülich GmbH, Peter Grünberg Institute (PGI-2) and JARA-FIT, 52425 Jülich, Germany; 7 Forschungszentrum Jülich GmbH, Peter Grünberg Institute (PGI-5) and JARA-FIT, 52425 Jülich, Germany; 8 Forschungszentrum Jülich GmbH, Peter Grünberg Institute (PGI-1) and JARA-FIT, 52425 Jülich, Germany

## Abstract

Three-dimensional (3D) topological insulators are a new state of quantum matter, which exhibits both a bulk band structure with an insulating energy gap as well as metallic spin-polarized Dirac fermion states when interfaced with a topologically trivial material. There have been various attempts to tune the Dirac point to a desired energetic position for exploring its unusual quantum properties. Here we show a direct experimental proof by angle-resolved photoemission of the realization of a vertical topological p–n junction made of a heterostructure of two different binary 3D TI materials Bi_2_Te_3_ and Sb_2_Te_3_ epitaxially grown on Si(111). We demonstrate that the chemical potential is tunable by about 200 meV when decreasing the upper Sb_2_Te_3_ layer thickness from 25 to 6 quintuple layers without applying any external bias. These results make it realistic to observe the topological exciton condensate and pave the way for exploring other exotic quantum phenomena in the near future.

## Abstract (web summary)

Topological insulators possess dispersionless electronic surface states with perpendicular spin-momentum locking which may be utilised in spintronic devices. Here, the authors demonstrate p–n junctions formed from two topological insulator thin films, tuning the junction type by film thickness.

## Graphical abstract

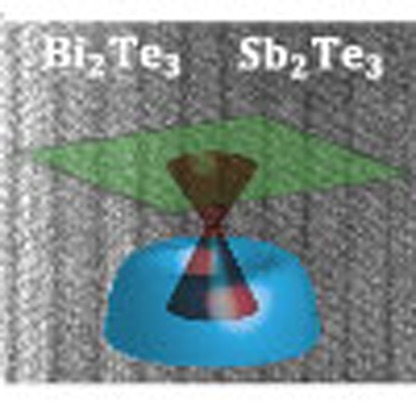

It is well-known that binary compounds X_2_Y_3_ based on high-Z elements with sufficiently strong spin-orbit coupling, such as X=Bi or Sb and Y=Te or Se, belong to the class of strong three-dimensional (3D) topological insulators (TIs). They host an odd number of gapless Dirac cone-like linear dispersive surface states with chiral spin-momentum locking, which are located around high symmetry points in the surface Brillouin zone^1^,^2^,^3^,^4^,^5^. From a practical point of view, these materials can be a basis for novel dissipationless spintronic devices because the propagation direction of their surface electrons is robustly locked to their spin orientation, that is, back-scattering in charge transport is prohibited as long as time-reversal symmetry is preserved^6^,^7^.

Studying the Dirac fermion states promises the verification of exotic quantum phenomena, such as the image magnetic monopole, which may exist in TIs due to proximity effects to a ferromagnetic material^8^, or Majorana fermions, which can be induced at the interface of a strong TI and an s-wave superconductor^9^. More specifically, the spatial separation of variable Dirac cone structures opens up the possibility to study what was proposed as the horizontal topological p–n junction^10^, where the effect of the lateral variation of the chemical potential on the spin-locked transport can be investigated, including its control by external electric fields^11^.

Moreover, the vertical (with respect to the sample plane) separation of the Dirac cones might enable the observation of the so-called topological exciton condensate, which is proposed to exhibit fractionally charged excitations (similar to Majorana fermions) in its vortices without any additional interface^12^. The only prerequisites are separated electron- and hole-type Dirac fermions on opposite surfaces which interact electrostatically.

There has been considerable amount of research to precisely tune the position of the Fermi level *E*_F_ in topological Dirac cones. First, this can be achieved by surface doping^13^ which, however, does not lead to a suppression of the bulk conductivity. Another successful path is to gradually tune the composition in a ternary^11^,^14^,^15^,^16^ (or even quaternary^16^) alloy, like (Bi_1−*x*_Sb_*x*_)_2_Te_3_. Since typical epitaxially grown layers of Bi_2_Te_3_ (Sb_2_Te_3_) turn out to be of n-(p-)type charge character, which are dominated by electron (hole) transport in the bulk^17^, this alloying leads to an effective compensation of charge and thus to a shift of the chemical potential and tunable surface states and eventually also suppression of the bulk conductivity.

Similarly, bringing together two different binary TI films to create a vertical topological p–n junction should also lead to compensation of charge within the depletion layer formed at their interface. However, the effect of such a topological p–n junction on the topologically protected surface states or the surface electronic structure in general has not been reported so far.

In this article, we present the direct observation of thickness-dependent electronic shifts of the chemical potential in vertical topological p–n junctions by means of angle-resolved photoemission spectroscopy (ARPES). The junctions are created in Sb_2_Te_3_/Bi_2_Te_3_ heterostructures of variable layer thickness, grown by molecular beam epitaxy, which assures high-crystalline quality and high accuracy of the thickness and composition of the thin films. The thickness of the underlying Bi_2_Te_3_ layer is kept constant for all samples as approximately six quintuple layers (QLs), whereas the Sb_2_Te_3_ layer thickness *t* is varied *t*=25, 15, 7, 6 QL. Modifying the top layer thickness results in a varying influence from the buried layer on the probed upper surface and thus to a Dirac point (DP) shifting up to 200 meV with respect to the Fermi level. In this way we are able to alter an Sb_2_Te_3_ surface from being of p-type charge carrier character to n-type by reducing the thickness above the Bi_2_Te_3_ layer.

Thus, we believe that our findings add a fundamentally new approach to the conventional ones, such as doping or biasing, to engineer the band structure in TIs and especially their Dirac cone by intrinsic interfacial effects. Further, we believe that such advanced synthesis techniques will allow for the study of the interaction of opposing Dirac cones and potential new quantum states such as the topological exciton condensate.

## Results

### Structural analysis

**Figure 1:**
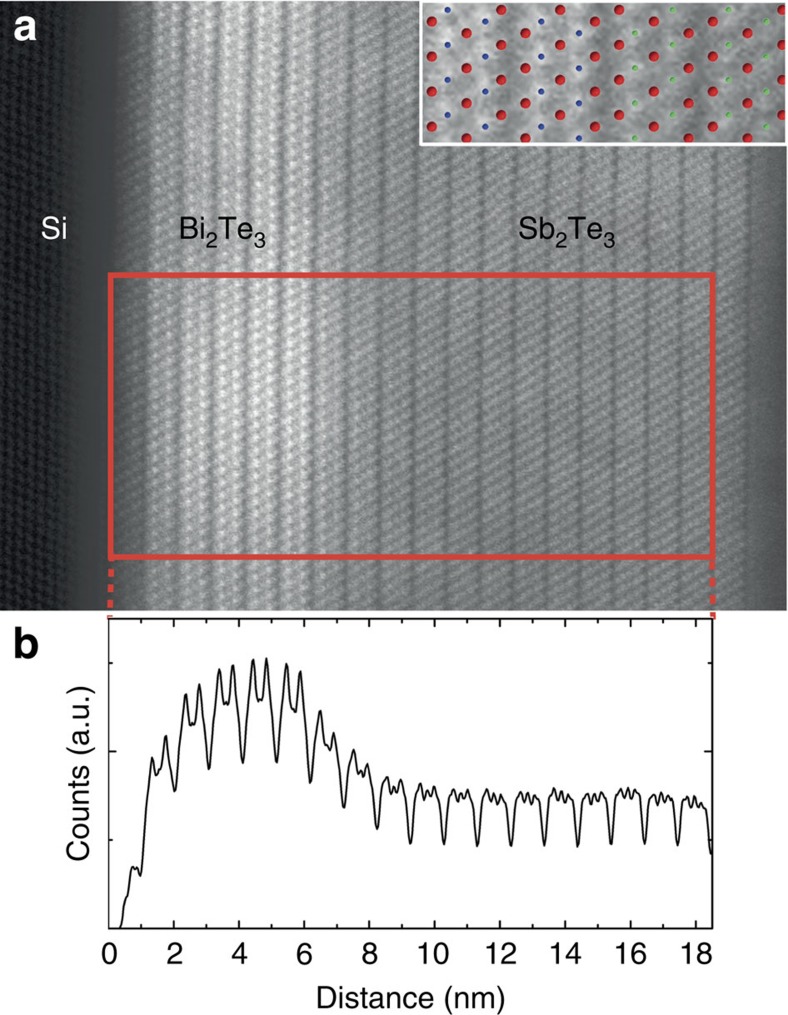
Structural analysis of the 15 QL Sb_2_Te_3_/6 QL Bi_2_Te_3_ sample via STEM. (**a**) HAADF image of atomic resolution. The large overview image reveals the high quality of the crystal. Van der Waals gap separated quintuple layers can be observed. The contrast in the image is related to the size of the atoms on which electrons are scattered, that is, chemical contrast is obtained. To estimate the size of the intermixed interface region a line profile is plotted in **b**, integrated over the red rectangle in **a**. This line profile also serves as a scale bar for **a**. The inset in **a** shows a magnified region across the interface of the two layers with a structural model superimposed (blue atoms, Bi; green atoms, Sb and red atoms, Te).

High-resolution scanning transmission electron microscopy (STEM) measurements were carried out to investigate structure and quality of the heterostructures. Figure 1a displays a high-angular annular dark field (HAADF) image of a 15 QL Sb_2_Te_3_/6 QL Bi_2_Te_3_ sample. According to the difference in atomic number, Bi atomic columns appear brightest. The crystalline quality and the degree of structural order of the individual QLs, which are clearly separated by van der Waals gaps, are very high. Only at the interface to the Si substrate, the contrast is slightly deteriorated due to amorphization during preparation of the sample for STEM (see Methods section) or the additional Te bilayer at the Si interface, which was reported by Borisova *et al.*^18^. This contrast change is also observed in very thin areas of the sample. Hence, only thick areas are suitable for investigation, which implies a loss of resolution. Nonetheless, the individual atomic columns are clearly revealed, which is highlighted in the inset displaying four QLs across the interface at higher magnification with a structural model as overlay. In Fig. 1b the intensity averaged within the red frame (in Fig. 1a) is plotted versus distance (also serving as scale of the STEM image in Fig. 1a). Towards the Si substrate, a decrease in counts is observed indicating a reduction in specimen thickness, which is in line with the observed amorphization. At the Sb_2_Te_3_/Bi_2_Te_3_ interface, the intensity is observed to decrease over a region of two QLs, that is, 2 nm. Since the contrast within the Sb_2_Te_3_ remains constant, we assume a constant thickness across the interface as well. Hence, the intensity gradient across the small interface region implies intermixing of Bi and Sb.

Since STEM is a very local probe, additional characterizations, such as low-energy electron diffraction (LEED) and Auger electron spectroscopy (AES) depth profiling, were performed. Supplementary Note 1 gives detailed information on AES depth profiling of the 15 QL Sb_2_Te_3_/6 QL Bi_2_Te_3_ sample, being in good agreement with our STEM data. Low-energy electron diffraction reveals the well-known sixfold diffraction pattern, indicating high-crystalline perfection of the top surface (Supplementary Fig. 1).

### Transport measurements

**Figure 2:**
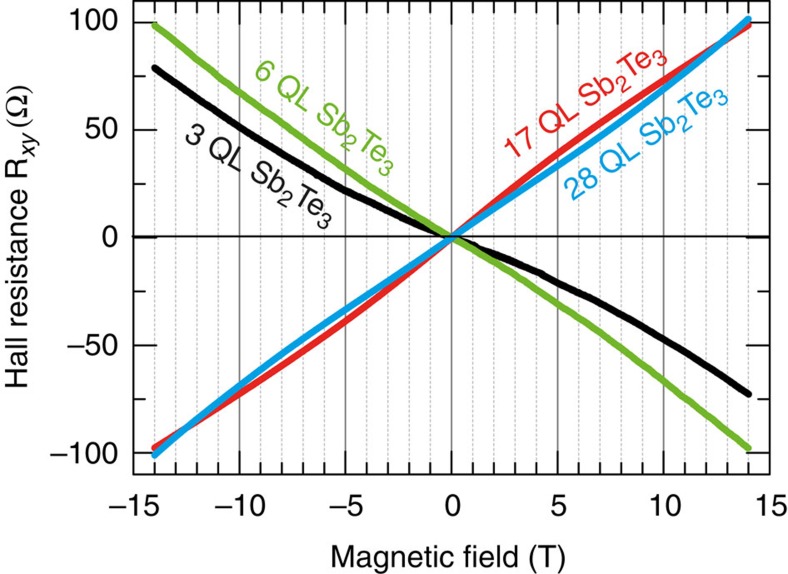
Field-dependent transport measurements. Hall resistance *R*_*xy*_ of four different samples with varying top Sb_2_Te_3_ layer thickness investigated at fixed gate voltages and low temperature. For thinner top layer thickness of 3 QL (black curve) and 6 QL (green curve), the heterostructure is in an n-type (electron) transport regime, whereas for thicker films of 17 QL (red curve) and 28 QL (blue curve) p-type (hole) transport is dominant.

High-quality MBE-grown Bi_2_Te_3_ films are known to exhibit mostly n-type charge carriers due to Te vacancies that introduce donors. Besides vacancies, also ionized Bi_Te_ antisite defects generate the n-type doping^17^,^19^. On the contrary, in Sb_2_Te_3_ the major defects are Sb_Te_ antisite defects, which impose p-type charge carriers. For this reason, Sb_2_Te_3_/Bi_2_Te_3_ heterostructures are expected to exhibit a separation of opposite carrier character making them a natural p–n junction system. To prove the existence of different regimes of charge carrier types, we performed magnetic field-dependent transport measurements at 1.4 K in standard Hall-bar geometry with sample widths between 20 and 40 μm and lengths between 150 and 300 μm.. The resulting Hall resistances are shown in Fig. 2.

We observe a transition from n- to p-type regime for increasing top Sb_2_Te_3_ layer thickness. The slope of the transversal Hall resistance *R*_*xy*_ changes from negative to positive between a sample thickness of 6 QL (green curve) and 17 QL Sb_2_Te_3_ (red curve). This implies that the electronic transport is mostly dominated by electron (n-type) and hole (p-type) transport. There exists a non-linearity of the Hall resistance at lower fields, which is interpreted as an effect due to the coexistence of both p- and n-type charge carriers in the samples. However, it is neither as strong nor it changes its slope as it was previously reported for certain gate voltages in similar heterostructures^20^. The slight change of the slope between the green (six QL sample) and black curve (three QL sample), which is opposite to the general trend, is due to the accuracy of this measurement.

### Electronic structure

**Figure 3:**
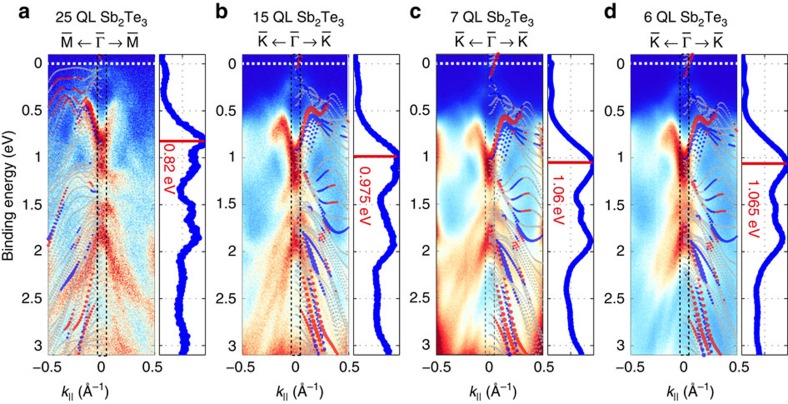
Wide energy *E*_B_ versus *k*_||_ ARPES maps. 25 QL (**a**) 15 QL (**b**) 7 QL (**c**) and 6 QL (**d**) Sb_2_Te_3_ samples measured along indicated crystallographic directions using *hν*=21.22 eV. The electronic structure of a 6 QL-thick Sb_2_Te_3_ slab calculated by DFT along the corresponding crystallographic direction is superimposed. Red and blue dots in this calculation refer to opposite in-plane spin orientation. The Fermi level is indicated by the white dashed line. The energy distribution curves (EDCs) which are integrated over the black dashed area are shown on the right of each ARPES map and mark the energetic position of the most prominent features. The main feature being the bottom of the lower Rashba-split surface state serves as a gauge for the observed energetic shift.

**Figure 4:**
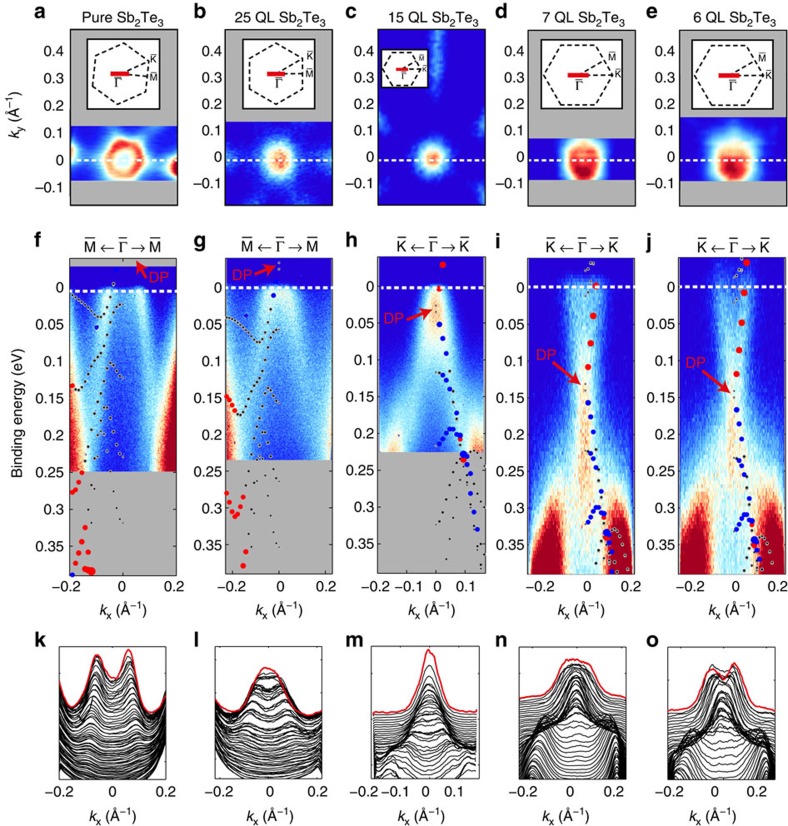
High-resolution ARPES close to the Fermi level using *hν*=8.44 eV. (**a**,**f**,**k**) present the results obtained for the reference single Sb_2_Te_3_ film. For the heterostructures, the Sb_2_Te_3_ top layer thickness is marked on top. **a**–**e** depict the measured Fermi surface maps *k*_*x*_ versus *k*_*y*_ for *E*_B_=*E*_F_. The black dashed lines in the insets depict the hexagonal shape of the surface Brillouin zone. This symmetry character is also conserved for the shape of the surface state as one departs from the Dirac point. The white dashed lines (red line in the inset) indicate the cut direction where the corresponding normal emission spectra (**f**–**j**) were recorded. The Dirac point is marked by red arrows and the band structure calculations from DFT with adopted Fermi energy are superimposed in each spectrum. Again, red and blue dots here represent opposite in-plane spin polarization of the states. **k**–**o** show the respective momentum distribution curves at binding energies from *E*_B_=0.2 eV (bottom) to *E*_B_=*E*_F_=0 eV (top, marked by the red line) of the spectra above.

The surface electronic structure of the studied heterostructures was mapped in detail using high-resolution ARPES. Figure 3 displays long scale *E*_B_ versus *k*_||_ ARPES maps along trajectories traversing the 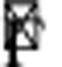-point of the surface Brillouin zone recorded with *hν*=21.22 eV. The exact cut directions in *k*-space were deduced by Fermi surface mapping and are highlighted in the insets of Fig. 4a–e. The plotted overview spectra all show dispersing bulk bands at relatively low background intensity, which signals the high-crystalline quality of the samples. Typical features of the Sb_2_Te_3_ band structure^21^ are revealed, such as the prominent Rashba-split surface state located between *E*_B_=0.4–0.8 eV and *k*_||_=±0.28 Å^−1^ in a spin-orbit induced gap within the projected band structure^22^. This feature is identified for all heterostructures. Furthermore, indications of the topologically protected Dirac cone states near the Fermi level are found in each spectrum. The photoemission cross-section for these states is small at 21.22 eV, however, they can be analysed in detail with lower photon energy (see next paragraph). An *ab initio* calculated electronic structure of a 6 QL-thick Sb_2_Te_3_ film along the corresponding crystallographic direction was superimposed on each spectrum to confirm the origin of the spectral features^21^.

The fact that all maps in Fig. 3 exhibit similar spectral features, which originate from pure Sb_2_Te_3_, allows to determine the energetic shift of the entire band structure towards higher binding energies for decreasing Sb_2_Te_3_ layer thickness. As highlighted by the respective energy distribution curves in Fig. 3, the bottom of the prominent Rashba-split surface state at 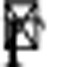, which has the largest spectral weight in these spectra, shifts by about 250 meV from 25 QL to 6 QL Sb_2_Te_3_.

The same effect of a shifting band structure is observed for the Dirac cone-like topological surface band near the Fermi level. Figure 4a–e presents the Fermi surface *k*_*x*_ versus *k*_*y*_ maps and Fig. 4f–j the *E*_B_ versus *k*_||_ spectra from a region close to the Fermi level for the pure Sb_2_Te_3_ film and the set of heterostructures (25 QL, 15 QL, 7 QL and 6 QL, respectively) measured at *hν*=8.44 eV. The magnified calculated electronic structure is superimposed in each spectrum. The insets in Fig. 4a–e illustrate the exact cut direction through the surface Brillouin zone. In addition, Fig. 4k–o depicts the corresponding momentum distribution curves.

In each of these high-resolution ARPES spectra, the Dirac cone can be observed. The spectra reveal that for decreasing Sb_2_Te_3_ top layer thickness the chemical potential of the sample surface is shifted from within the valence band through the forbidden band gap and towards the conduction band. Thereby, the DP crosses the Fermi level at about 15 QL Sb_2_Te_3_.

The samples consisting of pure Sb_2_Te_3_ (Fig. 4a,f,k) and the heterostructures with 25 QL (Fig. 4b,g,l) and 15 QL (Fig. 4c,h,m) Sb_2_Te_3_ top layer exhibit a Fermi level which still cuts the valence band. This is visible as sizable spectral weight from bulk bands with hexagonal symmetry within the Fermi surfaces. On the contrary, for the two thin films with 7 QL (Fig. 4d,i,n) and 6 QL (Fig. 4e,j,o) top layer thickness the Fermi level is well above the valence band and apparently inside the fundamental band gap with the DP below *E*_F_. This is in perfect agreement with the transport data shown in Fig. 2.

From the superimposed calculated electronic structure, the position of the DP with respect to the Fermi level is determined with an accuracy of ±20 meV. This method is known to be more precise than determining the intersection of two regression lines fitted to the Dirac cone^21^. The extracted binding energy positions of the DPs *E*_B_(DP) are listed in Table 1. The total energetic shift deduced from the shifting DP from the thickest to the thinnest heterostructure sample is about 200 meV, albeit slightly lower than the number derived from the wide energy spectra in Fig. 3. Furthermore, the Fermi velocity *v*_F_, derived from linear fits to the topological surface states close to the Fermi level according to *E*_B_=ℏ*v*_F_|*k*_||_|, is given in Table 1. Similar values have been obtained for pure Sb_2_Te_3_ and Bi_2_Te_3_ thin films in previous works^11^,^10^.

The observed energetic shifts of the electronic structure in ARPES and transport are highly reproducible and in accordance with the expected charge carrier character of the sample surface, which is of p-type or n-type depending on the distance to the Bi_2_Te_3_ layer.

### Comparison with 1D Schrödinger–Poisson model

Although there exist theoretical models which treat 3D TIs as highly doped narrow band gap semiconductors and question their ultimate bulk resistivity due to poorly screened random potential fluctuations^23^,^24^, recent experiments indicate that these limitations can be overcome in certain TI compounds^25^ or by the use of dual-gating of thin films^26^,^27^. Furthermore, Brahlek *et al.*^28^ show that band-bending effects indeed can lead to bulk insulating states in the Mott sense.

**Figure 5:**
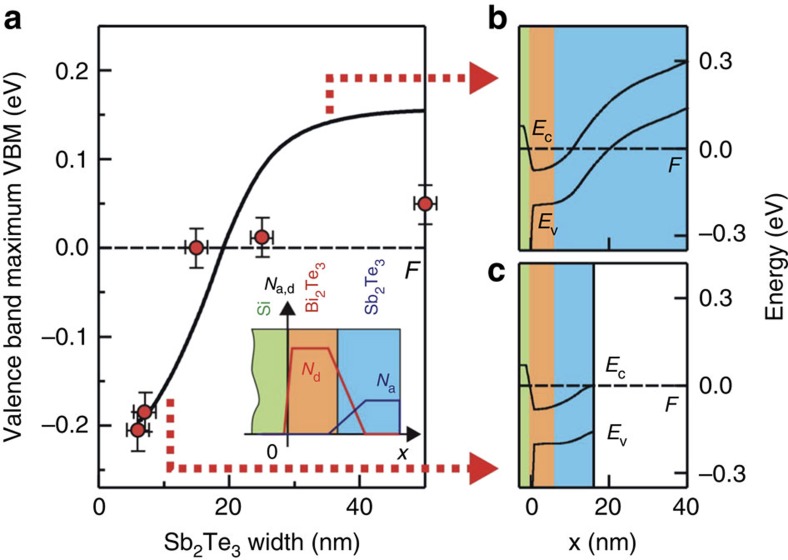
Result of the 1D model using Schrödinger–Poisson equation. (**a**) Calculated energetic position of the valence band maximum with respect to the Fermi level at the surface to vacuum for different Sb_2_Te_3_ layer thicknesses (black line) and experimentally derived values of the VBM from ARPES (red dots). A large error of ±25 meV was estimated on the position of the VBM because determination from ARPES is difficult. An error of ±2 nm was assumed on the accuracy of the film thickness from combined X-ray reflectivity and transmission electron microscopy investigations. The model assumes the creation of a depletion layer, which causes band bending. (**b**) and (**c**) show the band diagram of both valence and conduction band throughout the entire system for top layer thicknesses of 35 nm and 10 nm, respectively (connected by red dashed arrows to the curve in **a**). Green is the Si substrate, orange is Bi_2_Te_3_ and blue is Sb_2_Te_3_.

The latter makes us confident to be able to compare our results with a simulated band profile by modelling the TI heterostructure system for various thicknesses of the Sb_2_Te_3_ layer and self consistently solving 1D Schrödinger and 1D Poisson equations. The results of these simulations are shown in Fig. 5. Good agreement to the experimental data, considering the energetic position of the valence band maximum with respect to the Fermi level (Fig. 5a), is found if one assumes a donor-type (acceptor-type) charge carrier density of 2 × 10^19^ cm^−3^ (−2 × 10^18^ cm^−3^) in the Bi_2_Te_3_ (Sb_2_Te_3_) layer and an additional charge of 1 × 10^12^ cm^−2^ at the Bi_2_Te_3_/Si interface and the Sb_2_Te_3_ surface (all details of the model are given in Supplementary Note 2). To account for our knowledge of the slight intermixing at the interface, we also include into the model a 5-nm-wide interface region where the charge carrier density follows an experimentally deduced profile (by AES depth profiling, see Supplementary Fig. 1), which lead to further improvement of the comparison. Supplementary Fig. 2 additionally compares the result of the model for estimated limits of the carrier density, that is, for a perfectly sharp interface with zero intermixing on the one hand and very strong intermixing on the other hand.

Thus, the best agreement is found for slight intermixing of a 5-nm-wide interface region. Figure 5b,c exemplarily shows the band diagram of valence and conduction band throughout the entire system for Sb_2_Te_3_ thicknesses of *x*=35 nm and 10 nm, respectively. In the thin top layer regime (Fig. 5c), one can see that the samples are in an insulating state with the conduction band minimum close to the Fermi level, whereas for the thick top layers (Fig. 5b) the Fermi level cuts well through the valence band.

Finally, Fig. 5a summarizes the resulting position of the valence band maximum at the surface to vacuum plotted versus Sb_2_Te_3_ layer thickness. The dashed black line marks the Fermi level. The conduction band edge is not shown but would follow the same slope. In addition, the figure depicts the experimentally deduced values from the ARPES measurements (red dots). Experimentally, we observe a shift of the entire electronic structure of about 200 meV, while the simulations would predict a larger shift of about 350 meV. However, the trend is reproduced and thus the feasible agreement between our 1D model and our ARPES data again confirms that we have created a topological p–n junction.

## Conclusion

In conclusion, we have presented a new reliable way to precisely and robustly tune the charge carrier character and the chemical potential in 3D TIs by the creation of built-in interfacial electric fields in a vertical topological p–n junction. This method does not introduce disorder due to doping and does not require any external bias. Our heterostructures are a convenient playground for the study of Majorana fermions, which are predicted to be observed in tunnelling spectroscopy measurements of 3D TI-superconductor interfaces, if the DP is adjusted precisely at the Fermi level^9^,^29^. Moreover, Dirac electrons which reside in the (n-type) topological state at the interface between Bi_2_Te_3_ and silicon (which cannot be probed by ARPES) can couple to the (p-type) surface Dirac electrons such that a topological exciton condensate with similar properties as a topological superconductor is formed^12^. This new state of correlated quantum matter is so far elusive, but opens up a feasible alternative to probe effects like charge fractionalization in vortices or topological magnetoelectric effects.

In this respect, compared with dual-gating of a single TI layer, our approach of combining two binary TI layers in a p–n junction is much more versatile and, moreover, has the advantage of linearly dispersing Dirac states on both sides of the heterostructure, which enables the existence of two identical electron and hole Fermi surfaces. Consequently, the built-in spatial asymmetry of the Dirac bands and the reliable tunability of the chemical potential by manipulating purely internal structural parameters pave the way for studying exciting novel phenomena with potential applications in spintronics.

## Methods

### Growth

A set of epitaxial Bi_2_Te_3_/Sb_2_Te_3_ bilayers was grown by means of molecular beam epitaxy on high ohmic n-type doped Si:P(111) substrates of 10 × 10 mm size (doping level ∼10^13^ cm^−3^) under ultra-high vacuum conditions. Bi_2_Te_3_ is known to grow epitaxially on Si:P(111), forming films of high structural quality^30^. It grows in a rhombohedral structure with five atomic layers, known as QLs, as a basic unit forming relatively strong covalent bonds between the atoms within one QL, whereas the interaction among the consecutive QLs is of the van der Waals type. Since Bi_2_Te_3_ and Sb_2_Te_3_ have very similar lattice constants (Bi_2_Te_3_: *a*=4.385 Å, *c*=30.49 Å; Sb_2_Te_3_: *a*=4.264 Å, *c*=30.458 Å), Sb_2_Te_3_ grows epitaxially on Bi_2_Te_3_ as well. The growth rates were kept constant at 
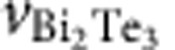
=11 nm h^−1^ and 
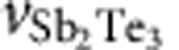
=9 nm h^−1^.

In addition to the set of Sb_2_Te_3_/Bi_2_Te_3_ bilayers, a 10-QL-thick Sb_2_Te_3_ film was prepared to serve as a reference sample. In those thickness regimes the coupling and hybridization of adjacent surface states through the layers can be neglected^31^,^32^. The detailed growth parameters, structural analysis and characterization of the system have been presented earlier^30^,^18^.

### ARPES

After growth the samples were transferred under ambient conditions into the high-resolution ARPES apparatus (base pressure <1 × 10^−10^ mbar). Subsequently, they were annealed up to 220–250 °C for 2 min to desorb surface contaminations. Afterwards the samples were cooled down and all measurements were carried out at ∼15 K. The spectra were taken with an MBS A1 analyzer set to an energy resolution of 10 meV for all presented ARPES data. The angular resolution in these ARPES experiments is <0.4°. Mapping of the electronic structure is achieved by rotating the sample around the axis, which is aligned along the long edge of the analyzer entrance slit.

Two different photon energies were used in these studies. To obtain overview spectra, non-monochromatized He Iα resonance radiation of *hν*=21.22 eV was employed since it allows access to the entire valence band (Fig. 3). Taking advantage of the large photoemission cross-section of Sb_2_Te_3_ surface states for low-energy photons^21^, monochromatized light from a microwave-driven Xe discharge lamp with *hν*=8.44 eV (Fig. 4) was used for the detailed analysis of the surface states.

### High-resolution STEM

Structural investigations on the atomic scale have been performed with an aberration-corrected STEM (FEI Titan 80–300) on cross-sectional specimen. The contrast of the HAADF images approximately scales with the atomic number *Z*^2^, which allows to distinguish between elements of large difference in atomic number, such as Sb or Te compared to Bi. Specimens have been prepared by focused ion beam etching using first 30 keV Ga ions followed by a 5 keV final treatment. Ar ion milling with the NanoMill operated at 900 V and subsequently at 500 V was employed to reduce the surface damage introduced by FIB.

### DFT calculations

All ARPES spectra presented here are compared with theoretical calculations of the electronic band structure of a six-QL-thick Sb_2_Te_3_ film. The theoretical spectra were calculated by full-relativistic density functional theory using the generalized gradient approximation and a full-potential linearized augmented plane wave method, which is implemented in the FLEUR code. For more details see ref. 33.

### 1D Schrödinger–Poisson model

See Supplementary Note 2.

## Additional information

**How to cite this article:** Eschbach, M. *et al.* Realization of a vertical topological p–n junction in epitaxial Sb_2_Te_3_/Bi_2_Te_3_ heterostructures. *Nat. Commun.* 6:8816 doi: 10.1038/ncomms9816 (2015).

## Supplementary Material

Supplementary Information Supplementary Figures 1-2, Supplementary Notes 1-2 and Supplementary References.

## Figures and Tables

**Table 1:** Dirac point binding energies and Fermi velocities at the surfaces of investigated heterostructures.

**Sample**	***E***_**B**_**(DP) (meV)**	***v***_**F**_ **(10**^5^** ms**^−1^)
Pure Sb_2_Te_3_	−65	4.4
25 QL	−35	2.5
15 QL	+30	2.2
7 QL	+140	5.2
6 QL	+145	4.8

Binding energy position of the Dirac point is extracted from the superimposed calculations. Negative (positive) binding energy refers to the unoccupied (occupied) band structure above (below) the Fermi level. Third column shows the Fermi velocities derived from linear fits to the surface states close to the Fermi level.
