# Supplementary material for: Realization of a vertical topological p–n junction in epitaxial Sb2Te3/Bi2Te3 heterostructures
Source: Nat Commun. 2015 Nov 17;6:8816. doi: 10.1038/ncomms9816 (PMC4660041; doi:10.1038/ncomms9816)
Supplement: Supplementary Information — Supplementary Figures 1-2, Supplementary Notes 1-2 and Supplementary References. [file ncomms9816-s1.pdf]

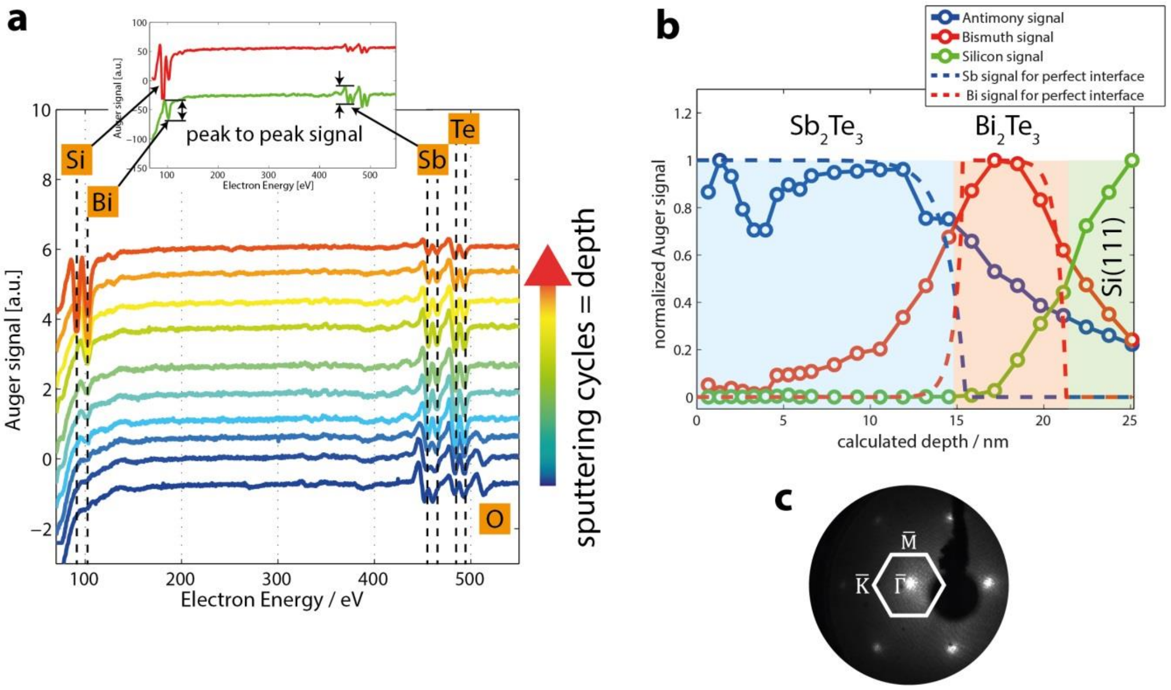

Supplementary Figure 1 | Structural analysis of the 15 QL Sb_2Te_3 / 6 QL Bi_2Te_3 sample via AES depth profiling. (a) Single AES spectra obtained after repeated cycles of ion sputtering (from blue = untreated to red). Peaks belonging to different elements are marked. The inset shows how the peak-to-peak signal was obtained. **(b)** The peak-to-peak signal from Bi (red), Sb (blue) and Si (green) is plotted against sputtering time which is recalculated into sample thickness by calibration via the STEM measurements. The dashed lines mark the lower limit of accuracy which this method can provide assuming an ideal sharp interface between the two materials. **(c)** Low-energy electron diffraction (LEED) pattern for this sample showing the high crystalline quality of the (111) surface.

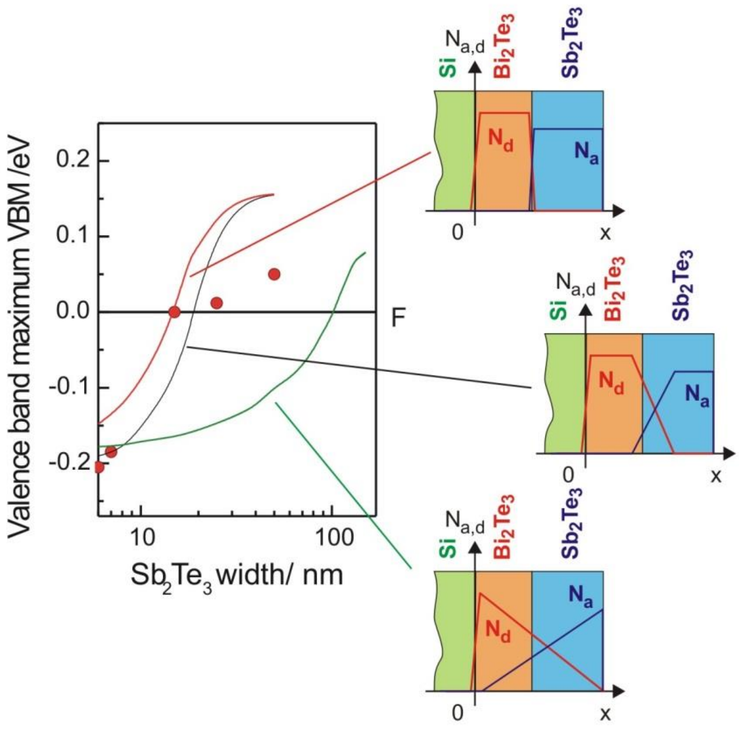

Supplementary Figure 2 | Estimation of the limits of our 1D model. The calculated energetic position of the valence band maximum is plotted against $\mathrm{Sb_2Te_3}$ top layer thickness. Experimental results (red dots) are compared to the results from our 1D Schrödinger Poisson model calculations. Red curve shows result for a perfectly sharp interface between the two $\mathrm{Bi_2Te_3}$ and $\mathrm{Sb_2Te_3}$ layers and the green curve, respectively, for a very strong gradual intermixing of charge carriers. The black curve assumes a light composition profile on the basis of the experimentally deduced profile (from Supplementary Fig. 1b). Each situation is schematically illustrated. Green is the Si substrate, orange the $\mathrm{Bi_2Te_3}$ layer whose thickness is fixed and blue the varying $\mathrm{Sb_2Te_3}$ layer. Red and blue lines here represent donor- and acceptor-type carrier distribution, respectively.

Supplementary Note 1

Composition profiling. Selected samples have been investigated by repeated gentle 500 eV $\mathrm{Ar^+}$ ion sputtering and subsequent Auger electron spectroscopy (AES) cycles in order to determine the composition profile. Supplementary Fig. 2 shows the result of such AES depth profiling for a 15 QL $\mathrm{Sb_2Te_3}$ / 6 QL $\mathrm{Bi_2Te_3}$ sample. From the single AES spectra presented in Supplementary Fig. 1a the relative amount of Bi, Te, Sb and Si was determined. Hence, the composition can be plotted against the sputtering cycle and, if one uses the known sample thickness from STEM (see Fig. 1), this can be recalculated into a real film thickness, as done in Supplementary Fig. 1b. Separated regions of $\mathrm{Sb_2Te_3}$ and $\mathrm{Bi_2Te_3}$ can be clearly distinguished but again a sizable diffusion of Sb and Bi at the interface is found which might have been induced by the additional annealing step. To be more precise, firstly, Sb diffuses to the $\mathrm{Bi_2Te_3 - Si}$ interface and, secondly, Bi shows a non-vanishing signal throughout the entire heterostructure. However, at the surface to vacuum the characteristic low energy 103 eV NOO Auger peak of Bi was found to be small in all samples. The depth profiling confirms the existence of an intermixed interface region between the two TI layers of width of a few nm. The real

extension of this region will be smaller than the red or blue profiles because one has to take several broadening effects into account.

Firstly, the resulting AES signal will be a convolution of the true profile and a thickness-dependent function that includes inelastic mean free paths of the Auger electrons (simulated by the dashed lines in Supplementary Fig. 1b). Such a function gives an upper limit of the accuracy of the AES profiling method, assuming that the sputtering process proceeds homogeneously, i.e. in the layer-by-layer mode. Secondly, the measured profile is convoluted with the depth resolution function [1], taking into account sputtering-induced changes in the composition and surface roughness, the assessment of which is beyond the scope of the present analysis. This means that the true profile is expected to be steeper than derived here, which is in good agreement with the presented STEM data and also means that our cleaning procedure did not significantly influence the quality of the interface.

Supplementary Note 2

1D Schrödinger-Poisson model. To estimate the band bending within the p-n junction created by the connection of two narrow band gap semiconductors with opposite dominating charge character, we modeled the system in 1D solving the conventional 1D Poisson and 1D Schrödinger equations self consistently [2]. The Schrödinger equation was written for the envelope function using the effective mass approximation. Numerically, both equations were iteratively solved and the solution was altered until the charge neutrality of the structure was fulfilled. The system consists of a semi-infinite Si-substrate, a 6 nm thick layer of n-type doped $\mathrm{Bi_2Te_3}$ and a layer of varying thickness of p-type doped $\mathrm{Sb_2Te_3}$ (Figure 5, inset). The electron and hole effective masses as well as the band gaps were taken from the results of ab-initio calculations [3, 4]. To our knowledge, the band offset between Si and $\mathrm{Bi_2Te_3}$ has so far not been determined and was assumed to be equal to the difference between the electron affinities of Si (4.05 eV from ref. [5]) and $\mathrm{Bi_2Te_3}$. The $\mathrm{Bi_2Te_3}$ electron affinity ranges from 4.125 to 4.525 eV (ref. [6]), thus the band offset value of 0.3 eV between Si and $\mathrm{Bi_2Te_3}$ seems acceptable. Values for the dielectric constant for both materials were found in literature to be $\varepsilon_{\mathrm{Bi_2Te_3}} = 75$ [7] and $\varepsilon_{\mathrm{Sb_2Te_3}} = 36.5$ [8]. Different numbers for the bulk native defects and surface states density were computed and the resulting band diagrams compared to experimental spectra. The values with best agreement were found to be for donor-type native defects in $\mathrm{Bi_2Te_3}$ - $N_\mathrm{d} = 2 \cdot 10^{19}\ \mathrm{cm^{-3}}$ and acceptor-type native defects in $\mathrm{Sb_2Te_3}$ - $N_\mathrm{a} = 2 \cdot 10^{18}\ \mathrm{cm^{-3}}$. Additionally, two layers of negative surface charge with a density of $1 \cdot 10^{12}\ \mathrm{cm^{-2}}$ were assumed at the interfaces between topologically trivial to non-trivial materials, i.e. interface of $\mathrm{Bi_2Te_3}$ to the Si substrate and at the $\mathrm{Sb_2Te_3}$ surface to vacuum. All these charge densities are within experimentally confirmed uncertainty limits. All calculations were performed assuming T = 20 K. Instead of an abrupt defect distribution step, an intermixed interface region of 5 nm width derived from Supplementary Fig. 1b was included in the model. The band gap within the two materials has been linearly changed from 0.12 eV for $\mathrm{Bi_2Te_3}$ to 0.16 eV for $\mathrm{Sb_2Te_3}$ within this region. The model calculates the band diagram through the entire sample but only the resulting valence band position at the surface to vacuum was plotted against top layer $\mathrm{Sb_2Te_3}$ thickness and compared to experimental ARPES results in Figure 5.

Estimations for extreme limits of the model.

In order to evaluate the effect of intermixing on the position of the energy bands, we modeled also for two extreme cases of no and very strong intermixing. Supplementary Fig. 2 again shows the position of the valence band maximum vs. $\mathrm{Sb_2Te_3}$ top layer thickness. The red dots and the black curve are the same as in Figure 5 and show experimental ARPES data and the results of the model for an intermixing which is based on the measured profile in Supplementary Fig. 1b. Additionally, the red curve depicts the results of the model for a perfectly sharp interface, i.e. no intermixing at all, and the green curve for strong gradual intermixing of the charge carrier density through the entire structure (the carrier density distribution is depicted on the right of Supplementary Fig. 2 for each

curve). As one can see, the green curve is quite different from our experimental results, whereas the red and black curve are similar, showing that strong intermixing would not match the ARPES and transport results.

References

[1] Briggs, D. & Seah, M.P. Practical Surface Analysis, Auger and X-ray Photoelectron Spectroscopy. Wiley. (1990)

[2] Stern, F. Iteration Methods for calculating self-consistent fields in semiconductor inversion layers. *J. Comput. Phys.*, **6**, 56-67 (1970).

[3] Nechaev, I. & Chulkov, E. Quasiparticle band gap in the topological insulator Bi2Te3. *Phys. Rev. B*, **88**, 165135 (2013).

[4] Yavorsky, B., Hinsche, N., Mertig, I. & Zahn, P. Electronic structure and transport anisotropy of Bi2Te3 and Sb2Te3. *Phys. Rev. B*, **84**, 165208 (2011).

[5] Ioffe Physico-Technical Institute. Physical Properties of Semiconductors. Electronic archive - New Semiconductor Materials. Characteristics and Properties. http://www.ioffe.rssi.ru/SVA/NSM/Semicond/Si/basic.html (2001)

[6] Nagao, J., Hatta, E. & Mukasa, K. Evaluation of metal - Bi_2Te_3 contacts by electron tunneling spectroscopy. Proceedings of the XV International Conference on Thermoelectrics. Pasadena, CA , USA, 404 (1996).

[7] Richter, W., Köhler, H. & Becker, C.R. A Raman and Far-Infrared Investigation of Phonons in the Rhombohedral $V_2 - VI_3$ Compounds. *Phys. Status Solidi (b),* **84**, 619 (1977).

[8] Drope, R. PhD thesis, RWTH Aachen University (1975).
